# Infections caused by extended-spectrum beta-lactamases producing *Enterobacteriaceae*: clinical and economic impact in patients hospitalized in 2 teaching hospitals in Dakar, Senegal

**DOI:** 10.1186/s13756-016-0114-7

**Published:** 2016-04-18

**Authors:** Awa Ndir, Amadou Diop, Roughyatou Ka, Pape Makhtar Faye, Ndeye Mery Dia-Badiane, Babacar Ndoye, Pascal Astagneau

**Affiliations:** PhD Program, Université Pierre Marie Curie, Paris, France; Institut Pasteur de Dakar, Epidemiology unit, Dakar, Senegal; Hôpital pour Enfants Albert Royer, Dakar, Senegal; Centre Hospitalier Universitaire de Fann, Dakar, Senegal; Infection Control Africa NetworK, Cape Town, South Africa; Universités Sorbonne Paris-Cité, Paris, France

**Keywords:** ESBL, *Enterobacteriaceae*, Antibiotic resistance, Impact, Economic, Africa, Cost-of-illness, Multistate model

## Abstract

**Background:**

Infections caused by extended-spectrum beta-lactamases producing *Enterobacteriaceae* (ESBL-E) are of major concern in clinical practice because of limited therapeutic options effective to treat them. Published studies showed that ESBL-E, widely spread in Europe, United States or Asia; are also frequent in Africa. However, the impact of ESBL-E infections is yet to be adequately determined in Sub-Saharan African countries, particularly in Senegal.

The aim of our study was to estimate the incidence rate of ESBL-E infections and to assess their clinical and economic impact in Senegal.

**Methods:**

Two retrospective cohort studies were conducted in patients hospitalized from April to October 2012. A classic retrospective cohort study comparing patients infected by an *Enterobacteriaceae* producer of ESBL (ESBL+) and patients infected by an *Enterobacteriaceae* non-producer of ESBL (ESBL-) was carried out for fatal outcomes. Besides, a retrospective parallel cohort study comparing infected patients by an ESBL+ and ESBL- versus uninfected patients was carried out for the excess LOS analyses. Multivariable regression analysis was performed to identify risk factors for fatal outcomes. A multistate model and a cost-of-illness analysis were used to estimate respectively the excess length of stay (LOS) attributable to ESBL production and costs associated. Cox proportional hazards models were used to assess the independent effect of ESBL+ and ESBL- infections on LOS.

**Results:**

The incidence rate of ESBL-E infections was 3 cases/1000 patient-days (95 % CI: 2.4–3.5 cases/1000 patient-days). Case fatality rate was higher in ESBL+ than in ESBL- infections (47.3 % versus 22.4 %, *p* = 0.0006). Multivariable analysis indicated that risk factors for fatal outcomes were the production of ESBL (OR = 5.7, 95 % CI: 3.2–29.6, *p* = 0.015) or being under mechanical ventilation (OR = 5.6, 95 % CI: 2.9–57.5, *p* = 0.030). Newborns and patients suffering from meningitidis or cancer were patients at-risk for fatal outcomes. ESBL production increased hospital LOS (+4 days) and reduced significantly the hazard of discharge after controlling for confounders (HR = 0.3, 95 % CI:0.2–0.4). The additional cost associated with ESBL-production of €100 is substantial given the lower-middle-income status of Senegal.

**Conclusion:**

Our findings show an important clinical and economic impact of ESBL-E infections in Senegal and emphasize the need to implement adequate infection control measures to reduce their incidence rate. An antibiotic stewardship program is also crucial to preserve the effectiveness of our last-resort antibiotic drugs.

## Background

Extended-spectrum beta-lactamases producing *Enterobacteriaceae* (ESBL-E) are of major concern since infections caused by these resistant strains are associated with prolonged hospital stay and increased case-fatality rate [1–3]. ESBL-E became a significant therapeutic challenge worldwide in daily clinical practice since their resistances to additional classes of antibiotics reduce effective therapeutic options [4–6]. However, the increased use of carbapenems, drugs of last-resort to treat these infections, favors the emergence of carbapenem-resistant *Enterobacteriaceae* [7–9]. It is therefore crucial to better understand the extent of the threat poses by ESBL-E and to quantify its burden in order to help policymakers and healthcare professionals to set priorities and implement effective countermeasures. In Africa published studies showed that ESBL-E prevalence rate is increasing and is varying from 0.7 % in Malawi to 75.8 % in Egypt [10, 11]. However, until now the burden of ESBL-E has not been clearly established especially in sub-Saharan African countries.

This study aimed to estimate the incidence rate of ESBL-E infections and to assess the clinical and economic consequences of these infections in two hospitals in Dakar, Senegal.

## Methods

### Study design and population

Two retrospective cohort studies were carried out in patients hospitalized from April to October 2012 at the University Hospital of Fann and Albert Royer Children’s hospital, 2 academic tertiary care hospitals located in the same geographic area in Dakar, Senegal (respectively a 339-Bed and 120-bed hospitals). A classic retrospective cohort study comparing patients infected by an *Enterobacteriaceae* producer of ESBL (ESBL+) and patients infected by an *Enterobacteriaceae* non-producer of ESBL (ESBL-) was carried out for fatal outcomes. Besides, a retrospective parallel cohort study comparing infected patients (ESBL+ or ESBL- infections) versus uninfected patients was carried out for excess LOS analyses. Patients with an infection caused by an *Enterobacteriaceae* strain were identified through a laboratory-based surveillance of strains recovered from diagnostic samples taken at least 48 h after the patient admission when an infection was suspected. The date of the infection onset was the date the first sample yielding an *Enterobacteriaceae* strain was collected. If a bacterial strain was isolated on several occasions, only the first isolation was considered. Uninfected patients were randomly selected from the hospital database system. Inclusion criteria for the group of uninfected patients were the absence of clinical signs of an infection reported in patients’ medical files, the absence of diagnostic samples drawn and antibiotics prescriptions during the hospital stay. Infected and uninfected patients were matched on ward and day of admission. All patients included in the study were followed from admission to discharge or in-hospital death. For each patient, the following variables were collected: gender, age, germs isolated and their resistance profile, underlying comorbidities, diagnostic at admission, interventions related to patient care before the infection onset, length of stay, date of infection onset, antibiotic prescriptions during the hospital stay and in-hospital mortality.

### Definitions

Newborns were defined as patients less than 28 days of life and included premature babies (babies born before 37 weeks of gestational age). Children were patients aged between 1 month and 16 years and adults were patients up to 16 years.

An infection was suspected if clinical signs of the infection were observed by the clinician and was bacteriologically confirmed when a bacterial strain was recovered from diagnostic samples. In newborns and children, an infection was suspected in the presence of fever (> = 38 °C), hypothermia (<36 °C) or other signs of infection as detailed in the World Health Organization (WHO) guideline of the Integrated Management of Childhood Illness [12]. Infections were defined as ESBL+ when the sample yielded an ESBL-producing *Enterobacteriaceae* (ESBL-E) and ESBL- when *Enterobacteriaceae* was non-producer of ESBL. Antimicrobial therapy was defined as empirical if prescribed initially before susceptibility tests results were available. Initial antibiotic therapy was considered as inadequate when the empirical antibiotic drug was not active against the pathogen causing the infection.

### Microbiological methods

*Enterobacteriaceae* strains were Gram strained and then identified with API 20E strips (bioMérieux, Marcy l’Etoile, France). Susceptibilities against antimicrobial agents and the detection of ESBL production were routinely performed in the two hospitals by the disc diffusion synergy method using discs containing cefepime, ceftazidime and cefotaxime each placed 30 mm apart around a disc containing clavulanic acid as recommended by the Antibiogram Committee of the French Microbiology Society [13].

### Statistical analysis

Continuous variables were compared using Student’s *t* test with unequal variances. Fisher’s exact test was used for the comparison of categorical variables.

To evaluate risk factors for fatal outcomes, a multivariable model was constructed using a backward stepwise logistic regression analysis including all variables with a *p*-value less than 0.20 in the univariate analysis. *P* value less than 0.05 was considered as significant.

### Multistate model

**Fig. 1:**
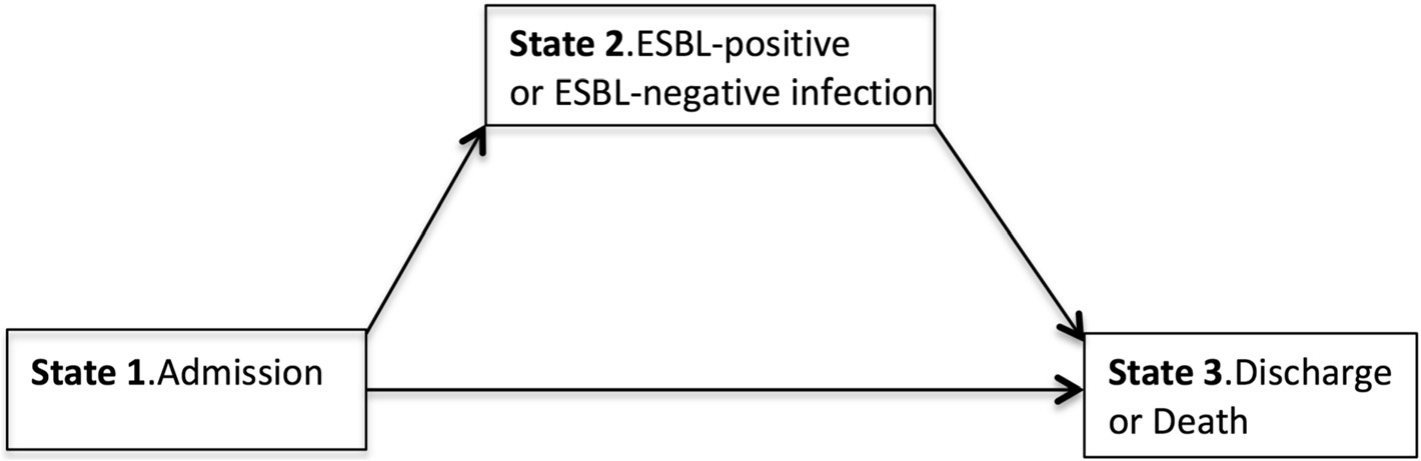
Multistate model used for the excess length of stay analysis. Every patient enters the model in state 1 on the day of admission, make a transition into state 2 at the time of infection (ESBL+ or ESBL-) then move to the 3 at the time of discharge or death. Uninfected patients enter in state 1 and move to state 3 without transition through state 2

The excess LOS attributable to ESBL production was estimated using a multistate model in which the occurrence of the infection was the time-dependent exposure, while the discharge (alive or dead) was the study endpoint (Fig. 1). Patients enter in the multistate model in state 1 at hospital admission. At the time of infection (ESBL+ or ESBL- infection) patients move to state 2 then to state 3 at the time of discharge or death. Patients who do not experience an infection during their hospital stay move directly from state 1 to state 3. The excess LOS attributable to ESBL production was the difference between LOS due to ESBL+ and ESBL- infections. When assessing the LOS due to ESBL+ infections, patients with an ESBL+ infection were compared to patients free of infection including uninfected patients and patients with an ESBL- infection but the latter were administratively censored at the time of infection. Likewise, patients with an ESBL+ infection were administratively censored when assessing the LOS due to ESBL- infection [14, 15].

Transition probabilities to move from one state to another were estimated non-parametrically using the Aalen-Johansen estimator [15–17]. The expected LOS was then computed by a function of the Aalen-Johansen estimator for the matrix of transition probabilities. The mean difference in LOS was computed for each day in the interval, as the difference between the estimated LOS given the intermediate state had been reached or not up that day. Standard errors and confidence intervals were calculated by 500 bootstrap resampling runs [15, 18, 19].

To assess the independent effect of ESBL+ and ESBL- infections on LOS, they were separately evaluated as time-dependent covariates using Cox proportional hazards modeling to estimate the end-of-LOS hazard ratio (HR). Variables for adjustment included age, comorbidities and invasive devices.

The R packages msm and etm were used for LOS analysis (version 2.15.3). All other statistical analyses were performed using Stata software, release 13.0.

### Cost-of-illness analysis

A cost-of-illness (COI) analysis was performed to quantify the cost associated with ESBL production from the patient perspective. Only direct medical costs incurred by patients from the date of the infection onset were measured. Indirect costs such as lost wages resulting from death or the absence from work were not considered in our analysis. A bottom-up approach was used to calculate the resources spent to treat the infection. Therefore total cost related to an infection (ESBL+ or ESBL+ infection) included costs of hospital stay, laboratory diagnostic tests and antibiotic drugs prescriptions. The cost of the hospital stay was calculated by multiplying the excess LOS attributable to the infection (estimated by multistate modeling) by the bed-day cost. For the costs related to diagnostic tests we considered the inclusive fee of €25 charged for all laboratory tests performed on a patient during the hospital stay, regardless of their number and nature. The dosage and duration of antibiotic drugs prescriptions to treat the infection were collected from patients’ medical files. The drug prices used for the calculations differ according to whether drugs were bought at the subsidized hospital pharmacy or purchased in private pharmacies.

### Ethical considerations

This study protocol was approved by the hospital’s institutional review board. Since the data were routinely collected and anonymized, ethical consent was not required.

## Results

### Characteristics of the study patients

During the 6-month study period, 186 *Enterobacteriaceae* strains were isolated in patients with a suspected infection. ESBL-E (n = 110) were *Klebsiella spp* (49.1 %), *Enterobacter spp* (28.2 %) and *E.coli* (22.7 %). The incidence rate of ESBL-E was 3 cases/1000 patient-days (95 % CI: 2.4–3.5 cases/1000 patient-days). The characteristics of the study patients are described in Table 1. The study population was constituted of 110 patients with an ESBL+ infection, 76 patients with an ESBL- infection and 186 uninfected patients. ESBL-E was most frequently associated with bloodstream infections (44.5 %), urinary tract infections (32.7 %) and surgical site infections (11.8 %) (Table 1).

**Table 1:** Baseline characteristics of patients infected by *Enterobacteriaceae* producer of ESBL (ESBL+) and *Enterobacteriaceae* non-producer of ESBL (ESBL-)

Variable, n (%)	ESBL+ infections (*n* = 110)	ESBL- infections (*n* = 76)	*P*-value
Demographics			
Male gender	68(61.8)	35(46.05)	0.037
Mean age, years (range)	26.3(20.9–31.7)	28.1(21.5–34.7)	0.665
Newborns (0–28 days)	22(20.0)	4(5.3)	0.005
Premature babies	12(10.9)	2(2.6)	0.046
Children (1 month-17 years)	37(33.6)	34(44.7)	0.167
Adults (up to 17 years)	51(46.4)	38(50.0)	0.656
Comorbidity			
Sickle cell disease	10(9.1)	7(9.2)	1
Malnutrition	10(9.1)	7(9.2)	1
AIDS	8(7.2)	2(2.6)	0.203
Diagnostic at admission			
Severe malaria	19(17.3)	7(9.2)	0.808
Gastroenteritis	16(14.5)	9(11.8)	0.666
Respiratory disease	25(22.7)	10(9.1)	0.127
Neurologic disorder	20(18.2)	12(15.8)	0.698
Meningitis	10(9.1)	5(4.5)	0.596
Tuberculosis	3(2.7)	3(3.9)	0.689
Cancer	10(9.1)	6(7.9)	1
Tetanus	4(3.6)	6(7.9)	0.321
Invasive procedure			
Surgical intervention	18(16.4)	18(23.7)	0.258
Parental nutrition	24(21.8)	6(7.9)	0.014
Mechanical ventilation	38(34.5)	7(9.2)	<0.0001
Central venous catheter	84(76.4)	35(46.0)	<0.0001
Urinary catheter	37(33.6)	13(17.1)	0.018
Type of infection			
Bloodstream infection	49(44.5)	20(26.3)	0.013
Urinary tract infection	36(32.7)	31(40.8)	0.279
Surgical site infection	13(11.8)	11(14.4)	0.656
Respiratory infection	5(4.5)	12(15.8)	0.016
Meningitidis	7(6.4)	2(2.6)	0.313
Time to infection, in days (95%CI)			
All patients	8.2(6.4–9.9)	4.8(2.7–6.9)	0.007
Adults	11.3(8.8–13.8)	7.7(4.8–10.6)	0.029
Children	6.6(3.1–10.2)	2(1.2–5.2)	0.028
Newborns	3.5(2.2–4.8)	1(0.3–2.3)	0.059
Mean time after infection, days (95 % CI)			
All patients	14.2(12.6–16.3)	9.3(7.4–11.1)	<0.0001
Adults	15.1(11.9–18.3)	8.9(7.2–10.6)	0.001
Children	15.5(12.3–18.7)	10(6.3–13.7)	0.013
Newborns	11.1(8.7–13.5)	6.2(5.8–13.0)	0.050
Inadequate antibiotherapy prescription	97(88.2)	14(18.4)	<0.0001

### Fatal outcomes

Sixty-nine patients with an infection caused by an *Enterobacteriaceae* died during the study period (37.1 %). The case-fatality rate was significantly higher in ESBL+ (47.3 %) than in ESBL- infections (22.4 %) (*p* = 0.0006). Patients who died were most frequently newborns, premature babies, patients who suffered from meningitidis or cancer, patients with an ESBL+ infection and patients with invasive devices such as mechanical ventilation or central venous catheter (Table 2).

**Table 2:** Risk factors for fatal outcomes: results of univariate and multivariable analyses

Variable, *n* (%)	Fatal outcomes		Univariate analysis			Multivariable analysis		
	Yes (*n* = 69)	No (*n* = 117)	OR	95 % CI	*P*-value	OR	95 % CI	*P-*value
Demographics								
Male gender	34(49.3)	49(41.9)	1.3	0.7–2.4	0.361			
Mean age, years (range)	27.1(20.4–33.8)	26.9(21.6–32.3)			0.489			
Newborns (0–28 days)	16(23.2)	10(8.5)	3.2	1.4–7.5	0.008	4.5	2.4–37.8	0.025
P remature babies	9(13.0)	5(4.3)	3.4	1.1–9.9	0.042			
Children (1 month–17 years)	17(24.6)	54(46.1)	2.6	1.4–5.0	0.005			
Adults (up to 17 years)	36(52.2)	53(45.3)	1.3	0.7–2.4	0.448			
Comorbidity								
Sickle cell disease	6(8.7)	11(0.5)	0.5	0.3–2.5	1			
Malnutrition	4(5.8)	13(11.1)	0.5	0.5–1.5	0.296			
AIDS	3(4.3)	7(5.9)	0.7	0.2–2.6	0.747			
Diagnostic at admission								
Gastroenteritis	6(8.7)	19(16.2)	0.5	0.2–1.3	0.184			
Respiratory disease	18(26.1)	17(14.5)	2.1	0.9–4.3	0.079			
Meningitis	11(15.9)	4(3.4)	5.3	1.7–16.6	0.004	2.3	1.5–4.9	<0.0001
Tuberculosis	2(2.8)	4(3.4)	0.8	0–4.1	1			
Cancer	12(20.3)	4(3.4)	5.9	1.9–18.3	0.002	2.7	1.6–6.6	0.001
Neurologic disorder	8(11.5)	24(20.5)	0.5	0.2–1.2	0.159			
Invasive procedure								
Surgical intervention	15(21.7)	21(17.9)	1.3	0.6–2.6	0.567			
Parenteral nutrition	15(21.7)	15(12.8)	1.9	0.9–4.1	0.148			
Mechanical ventilation	28(40.5)	17(14.5)	3.9	1.9–7.9	0.0002	6.1	3.1–124.6	0.040
Central venous catheter	53(76.8)	66(56.4)	2.6	1.3–4.9	0.007			
Urinary catheter	14(20.3)	36(30.8)	0.6	0.3–1.1	0.128			
ESBL Production	52(75.4)	58(49.6)	3.1	1.6–5.9	<0.0001	5.3	3.1–19.9	0.008
Type of infection								
Bloodstream infection	31(44.9)	38(32.5)	1.7	0.9–3.1	0.116			
Urinary tract infection	18(26.1)	49(41.9)	0.5	0.2–0.9	0.039			
Meningitidis	7(10.1)	2(1.7)	6.5	1.5–25.7	0.014			
Surgical site infection	9(13.0)	15(12.8)	1.0	0.4–2.4	1			
Respiratory infection	4(6.8)	8(7.7)	0.8	0.2–2.7	1			
Inadequate antibiotherapy prescription	49(71.0)	62(52.9)	2.3	1.2–4.3	0.013			
Mean time to infection, days, (95 % CI)	5.9(4.3–7.5)	7.3(5.4–9.3)			0.838			

Multivariable analysis showed that the production of ESBL (OR = 5.3, 95 % CI: 3.1–19.9, *p* = 0.008) or being under mechanical ventilation (OR = 6.1, 95 % CI: 3.1–124.6, *p* = 0.004) were independent risk factors for fatal outcomes. Results also showed that newborns and patients suffering from meningitidis or cancer were patients at-risk for fatal outcomes (Table 2).

### Length of stay (LOS)

Then mean LOS associated with ESBL+ and ESBL- infections was respectively 22.6 days (95 % CI: 20.3–24.9 days) and 14 days (95 % CI: 11.9–16.2 days). The mean time to infection was significantly longer in patients with an ESBL+ than an ESBL- infection except in newborns (Table 1). However, when comparing patient’s groups, the mean time to infection was shorter in newborns (3.5 days) than in children (6.6 days) or adults (11.3 days). Besides, the hospital stay after the infection was significantly longer in ESBL+ than in ESBL- infections (14.4 versus 9.3, *p* < 0,0001) suggesting that ESBL production prolonged the hospital LOS. Results presented in Table 3 indicated that ESBL production was associated with an excess LOS of 4 days (95 % CI: 3.8–4.6) and decreased significantly the hazard of discharge after adjustment for confounding (HR:0.3, 95 % CI:0.2–0.4). However, ESBL- infections did not decrease significantly the hazard of being discharged (HR:0. 9, 95 % CI:0. 7–1.2).

**Table 3:** Estimation of the excess length of stay (LOS) and hazard ratios (HR) of discharge associated with ESBL+ and ESBL- infection

		HR of discharge	
	Excess LOS, days (95 % CI)	Univariate (95 % CI)	Multivariable (95 % CI)
ESBL+ infection^a^	7.9 (7.6–9.2)	0.3 (0.2–0.4)	0.3 (0.2–0.4)
ESBL- infection^b^	3.9 (3.8–4.6)	0.9 (0.7–1.2)	0.9 (0.7–1.2)

*CI* confidence interval

^**a**^Model A: Excess LOS due to ESBL+ infection

110 patients with ESBL+ infection versus 76 patients with ESBL- infection censored at time of infection and 186 uninfected patients

^**b**^Model B: Excess LOS due to ESBL- infection

Seventy-six patients with ESBL- infection versus 110 patients with ESBL+ infection censored at time of infection and 186 uninfected patients

### Cost-of-illness analysis

The cost-of-illness (COI) analysis indicated a mean hospital cost significantly greater in ESBL+ (€215, 95 % CI: €196–233) than in ESBL- infections (€115, 95 % CI: €103–123), *p* < 0.0001. Thus, the additional cost attributable to ESBL production was €100 (95 % CI: €78–€125). Hospital stay and antibiotic drugs accounted respectively for 60 and 40 % of this cost.

## Discussion

Our study quantifies the economic impact of ESBL-E infections in patients admitted in 2 hospitals in Senegal. The additional cost of €100 attributable to the ESBL production may appear as low compared to costs reported in high-income countries, which vary from €5, 000 to €14, 720 [1, 2, 20, 21]*.* The difference in costs could be explained firstly by the high cost of equipment used in high-income countries, and secondly by the variability in methods used to estimate the economic impact of ESBL-E infections. Indeed, the occurrence of the infection considered as time-fixed in studies that compare infected patients with uninfected patients and the use of median values have been demonstrated to overestimate costs [22]. We avoided those pitfalls in our cost-of-illness (COI) analysis by comparing patients with ESBL+ and ESBL- infections versus uninfected patients, by considering the infection as time-dependent and measuring mean values. Costs were estimated from the patient’s perspective and not from the hospital or third-party payer perspective as frequently found in the literature. Indeed in Senegal, hospital costs are most of the time entirely outlaid by patients since only 20 % of the population has a healthcare coverage. Costs associated with ESBL+ or ESBL- infections might be considerable for some patients, since these costs are respectively 2.5-times and 1.3-times higher than the mean monthly salary (€87) in the country [23]. These out-of-pocket expenses can be crippling for already-impoverished households, pushing them further into poverty [24, 25]*.* Kruk et al. demonstrated that 37.8 % (95 % CI: 30.7–45.5) of Senegalese households often resort to “hardship financing” defined as borrowing money from family, friends or selling their assets to cover hospital costs [26]*.*

Besides, the hospital charges attributable to ESBL production would be more burdensome if antimicrobial therapy such as imipenem was prescribed. A 7-day treatment by imipenem would lead to an additional cost of €400. The choice of appropriate antibiotic drugs is crucial to treat ESBL-E infections; however, hospital stay was found to be the major driver of costs and could represent up to 60 % of costs as previously shown [21]. The time-dependent nature of infections should be taken into account when assessing the excess LOS attributable to a multidrug resistant pathogen. Indeed, it has been shown that the time-dependent bias will inevitably overestimate the impact of the infection on LOS and consequently on hospital costs [27, 28]. Therefore, we used a multistate model that accounts for time-varying exposure thereby avoiding the time-dependent bias inherent in other commonly used statistical methods [15, 28–30]. Results of the multistate model revealed an excess LOS attributable to ESBL production of 4 days (95 % CI: 3.8–4.6). Results of the Cox proportional hazards models showed a significant reduction in hazard of discharge associated with ESBL+ infections after adjustment on confounders and consequently a prolonged hospital stay.

Furthermore, the high rate of inadequate initial antimicrobial therapy (IIAT) in ESBL+ infections might delay the initiation of adequate therapy and consequently explain the prolonged LOS and the significant impact of ESBL+ infections on fatal outcomes demonstrated in our study [31, 32]. Indeed, the unavailability of imipenem in pharmacies or its unaffordability to the majority of patients may increase the risk of therapeutic failure in severe ESBL+ infections and thereby the risk of fatal outcomes. Our results suggest to adapt the current antibiotic prescriptions’ guidelines to the local bacterial epidemiology and to restrict the frequent empirical use of 3^rd^ generation cephalosporins.

The multistate model can be considered as a suitable approach when assessing the excess LOS attributable to ESBL production. However, while taking into account the time-dependent bias, the excess LOS may still be biased by other covariates. Besides, our findings should be interpreted with cautious since we could not collect data on prior antibiotic exposure that may potentially underestimate the incidence rate of ESBL+ infections. Additionally our results cannot be generalized to the whole hospitals in Senegal since our study was carried out only in two hospitals.

Despite these limitations, our findings should increase the awareness of healthcare decision makers on the threat poses by ESBL-E infections. Besides, these results should incite them to implement adequate infection prevention and control policies to reduce the incidence rate and the clinical and economic impact of ESBL-E infections. The assessment of the level of hand hygiene practices realized during the study period in the two hospital settings using a WHO tool [33] showed a basic level of hand hygiene suggesting that some measures were in place but not to a satisfactory standard. An infection control program including the promotion of alcohol-based handrub (ABHR) use should be implemented in these hospitals in order to improve their hand hygiene levels and to decrease the incidence rate of ESBL-E infections. Indeed, hand washing using alcohol-based handrub (ABHR) has been shown to be successful to reduce the incidence rate of antimicrobial resistant pathogens [34–36]. To ensure the availability of expensive ABHR in resource-poor countries, the WHO African Partnerships for Patient Safety program have launched a program in the two study settings for the local production of ABHR [37]*.* Additionally, since the human digestive tract is the main reservoir of *Enterobacteriaceae* strains, measures specifically targeting ESBL-E, such as the management of excreta, should also be implemented.

## Conclusion

We show that ESBL-E are frequent in Senegal and that infections caused by these multiresistant pathogens increase the case-fatality rate and the hospital length of stay of hospitalized patients and consequently have an important financial impact for patients. Our findings are a call for action to healthcare policymakers to consider multidrug resistance as a national health priority and to implement adequate infection control measures to tackle this worldwide issue. Surveillance surveys should be implemented in Sub-Saharan African countries in order to have a more accurate estimate of the global extent of ESBL-E, which threaten the effectiveness of our last-resort drugs.
